# T142. PARIETAL CONNECTIVITY IN SCHIZOPHRENIA AND PSYCHOTIC BIPOLAR DISORDER: A COMBINED STRUCTURAL AND DYNAMIC FUNCTIONAL CONNECTIVITY STUDY

**DOI:** 10.1093/schbul/sby016.418

**Published:** 2018-04-01

**Authors:** Tushar Das, Peter Liddle, Lena Palaniyappan

**Affiliations:** 1 University of Western Ontario; 2 University of Nottingham

## Abstract

**Background:**

The role of parietal lobe in psychotic disorders is poorly understood. In independent previous studies, we have observed that (1) the severity of disorganization is associated with reduced cerebral blood flow to bilateral parietal angular gyrus in patients with schizophrenia (2) disorganization is more pronounced in patients who have morphological abnormalities in left parietal supramarginal gyrus (3) the global connectivity of right parietal supramarginal region is reduced in schizophrenia compared to bipolar disorder with psychosis. We aimed to delineate the nature of parietal dysconnectivity in the 2 major psychotic disorders and to study the relationship between the syndrome of disorganization and structural and functional connectivity of the parietal lobe with rest of the brain. We also related parietal connectivity to the global assessment of functional status (GAF scores) and processing speed scores among patients with schizophrenia.

**Methods:**

We recruited 16 subjects with psychotic bipolar disorder and 34 subjects with established schizophrenia, age- and sex- matched with 32 healthy controls. Both patient groups were medicated, and were in a clinically stable state. Diffusion Tensor Imaging (DTI) and resting state fMRI data were obtained using a 3T MRI scanner. Using 90 regions as defined in the AAL atlas, deterministic tractography was performed (FSL v5.0 and TrackVis). For each of the 90*90 connections, fractional anisotropy weighted by number of streamlines, and normalised by average fiber length was used as the index of structural connectivity. 90*90 functional connectivity values were also obtained for each subject using the fMRI data (SPM8 and DPARSFA). Dynamic connectivity (variance) was estimated using a sliding window approach (13 bins; 240 time points; TR=2.5s). The primary variable of interest across the 2 imaging modalities was the graph metric of weighted average degree from all parietal lobe nodes in the AAL atlas with all other nodes of the brain. Using ANOVA, we compared the degree of parietal connectivity among the 3 groups of subjects. Three multiple regression analyses were conducted to assess relationships between parietal connectivity (degree of right and left structural and dynamic functional connectivity) and severity of disorganisation, processing speed (digit symbol substitution test -DSST) and GAF scores.

**Results:**

The 3 groups differed significantly on the degree of left parietal structural connectivity (F=6.5, p=0.002, HC>BIP=SCZ) and on the degree of left (F=6.4, p=0.003; BIP=HC>SCZ) as well as right parietal (F=5.2, p=0.008; BIP=SCZ>HC) dynamic functional connectivity. Parietal dysconnectivity predicted the severity of disorganisation (model F=4.1, p=0.01) in SCZ. Disorganization was particularly associated with reduced left parietal structural (β=-0.45, p=0.02) and dynamic connectivity (β=0.40, p=0.04) but not with the right parietal dysconnectivity. DSST scores were associated with reduced left parietal structural connectivity (β=0.44, p=0.04). GAF was increased in patients with higher right parietal dynamic functional connectivity (β=0.38, p=0.04).

**Discussion:**

Both structural and dynamic functional parietal dysconnectivity are seen in the 2 major psychotic disorders - schizophrenia and bipolar disorder. Left-right asymmetry in parietal dysconnectivity is notable, especially among patients with schizophrenia. Parietal dysconnectivity plays a role in the processing speed as well as global functioning deficits in schizophrenia. Taken together, these findings suggest that the degree of connectivity of parietal lobe could be an important determinant of symptom burden, specific cognitive deficits as well as functional capacity in psychotic disorders.

